# Cardiac resynchronization therapy: a comparison among left ventricular bipolar, quadripolar and active fixation leads

**DOI:** 10.1038/s41598-018-31692-z

**Published:** 2018-09-05

**Authors:** M. Ziacchi, I. Diemberger, A. Corzani, C. Martignani, A. Mazzotti, G. Massaro, C. Valzania, C. Rapezzi, G. Boriani, M. Biffi

**Affiliations:** 1 Institute of Cardiology, Department of Experimental, Diagnostic and Specialty Medicine, University of Bologna, Policlinico S.Orsola-Malpighi, Bologna, Italy; 2 Cardiology Division. Department of Diagnostics, Clinical and Public Health Medicine, University of Modena and Reggio Emilia, Policlinico di Modena, Modena, Italy

## Abstract

We evaluated the performance of 3 different left ventricular leads (LV) for resynchronization therapy: bipolar (BL), quadripolar (QL) and active fixation leads (AFL). We enrolled 290 consecutive CRTD candidates implanted with BL (n = 136) or QL (n = 97) or AFL (n = 57). Over a minimum 10 months follow-up, we assessed: (a) composite technical endpoint (TE) (phrenic nerve stimulation at 8 V@0.4 ms, safety margin between myocardial and phrenic threshold <2V, LV dislodgement and failure to achieve the target pacing site), (b) composite clinical endpoint (CE) (death, hospitalization for heart failure, heart transplantation, lead extraction for infection), (c) reverse remodeling (RR) (reduction of end systolic volume >15%). Baseline characteristics of the 3 groups were similar. At follow-up the incidence of TE was 36.3%, 14.3% and 19.9% in BL, AFL and QL, respectively (p < 0.01). Moreover, the incidence of RR was 56%, 64% and 68% in BL, AFL and QL respectively (p = 0.02). There were no significant differences in CE (p = 0.380). On a multivariable analysis, “non-BL leads” was the single predictor of an improved clinical outcome. QL and AFL are superior to conventional BL by enhancing pacing of the target site: AFL through prevention of lead dislodgement while QL through improved management of phrenic nerve stimulation.

## Introduction

Cardiac resynchronization (CRT) is a proven heart failure therapy, but a minority of patients (pts) have no clinical benefit^1^. The lack of improvement is multi-faceted, owing both to pts selection and technical CRT issues. Phrenic nerve stimulation (PNS), high myocardial threshold (HMT), left ventricular lead dislodgement (LD) and failure to achieve the target pacing site are the most frequent technical issues^1,2^, that were aimed at by the introduction of new left ventricular leads such as the quadripolar (QL) and the bipolar active fixation leads (AFL)^3–6^. This is the first study comparing 3 different LV lead platforms for CRT with defibrillator: bipolar passive fixation lead (BL), QL and AFL. The purpose was to evaluate at long term the performance of these 3 different LV leads from both the technical and clinical outcome standpoint.

## Methods

**Figure 1:**
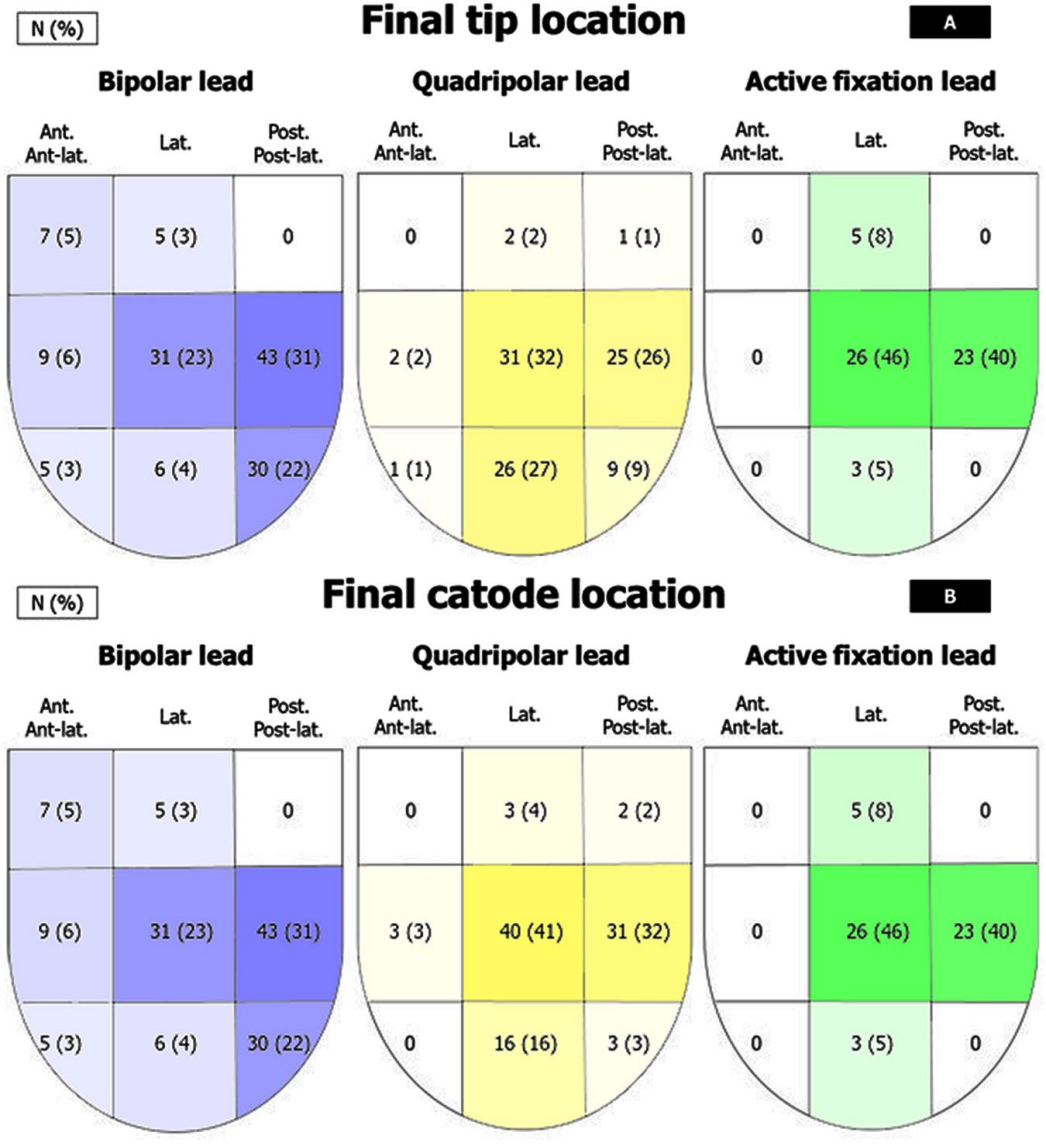
Final tip (**A**) and catode (**B**) locations of LV leads.

This was a single center observational study carried out on pts consecutively implanted with a CRT with defibrillator (D) and 3 different LV lead platforms: BL, QL and AFL. The study of LV performance was approved by the local Ethic Committee of the University Hospital S.Orsola-Malpighi (Bologna, Italy) and complies with the principles outlined in the Declaration of Helsinki. Pts provided informed consent for data collection and analysis. All the 4 implanting physicians have ≥10 years experience in CRT implantation, hence a learning-curve effect was excluded. The study enrolled all the pts implanted with CRT-D from January 2012 to June 2015. Pts with life expectancy <12 months were excluded. BL were implanted from January 2012 to December 2013; QL from June 2012 to June 2015, bipolar AFL were implanted from September 2013 to June 2015 (when they became available). Since December 2013 onward any bipolar lead implanted was an AFL. During the implant procedure an angiogram (obtained with an occlusive catheter) was taken in 45° left anterior oblique view (LAO) and in 30° right anterior oblique view (RAO). The target pacing site (TPS) was defined as the site with the longer Q-LV among all accessible veins^7^ sized at least 5 F. Preoperative imaging helped the selection process: areas of scarred tissue as detected by history of previous MI, echocardiography, SPECT, MR scan, were avoided to ensure stimulation of viable LV tissue, that predisposes to CRT non-response^8^. Q-LV was firstly measured at the great cardiac vein placement, then in the other coronary veins that had a minimum 5 F diameter (as showed by the angiogram), starting from the easiest accessible and ending with the more challenging one. Only coronary veins having a ratio Q-LV/total QRS duration ≥0.7 were considered suitable for the TPS. Thus, a TPS was achieved when this ratio was obtained by any available LV electrode^7^. When it became available, an AFL was preferred in the presence of a suitable coronary vein leading to posterior or lateral or antero-lateral site whose length was not exceeding 4 cm, whereas a QL was chosen for longer veins. LV leads location is shown in Fig. 1. Pacing configuration (LV-only vs Biventricular), atrioventricular and interventricular delay optimization occurred before discharge in all patients. “LV only” pacing configuration means LV stimulation avoiding right ventricle stimulation in the event of a normal intrinsic PR interval. The atrioventricular and interventricular optimization was guided by ECHO with the iterative method.

LV myocardial threshold, pacing impedance and phrenic nerve threshold were evaluated before discharge and at follow-up (in all pacing vector configurations). All the pts were evaluated clinically before implantation, at hospital discharge, and after at least 10 months of continuous resynchronization therapy.

We assessed composite technical endpoint (TE), clinical endpoint (CE), and LV reverse remodeling (RR) by Echocardiography.

### Definition of the composite TE

PNS at 8 V@0.4 ms in at least one pacing configuration (all the programmable LV pacing configuration were tested); safety margin between myocardial and phrenic threshold <2 V in the chosen pacing configuration not correctable with pacing vector reprogramming; left ventricular lead dislodgement requiring a re-operation and correctable with pacing vector reprogramming (variation of myocardial threshold >1.5 V suggested a possible LV lead dislodgement that need to be confirmed with an X-ray in antero-postero and latero-lateral view); failure to achieve the TPS.

### Definition of composite CE

death for any cause, hospitalization for heart failure and heart transplantation after at least 10 months of resynchronization therapy; infection leading to lead extraction.

RR (reduction of end systolic volume >15% compared to the value before the implant) was evaluated by the echocardiography according to Ypenburg classification^9^ after 10 months of continuous CRT (percentage of pacing >99%). The reproducibility of measurements in our center has been previously reported^10^.

## Statistical Analysis

### Descriptive statistics are reported as mean ± standard deviation (SD) for normally distributed variables

Categorical variables are reported as percentages and were statistically tested by means of the χ^2^. Freedom from clinical and technical endpoints was studied by means of the Kaplan–Meier method. Univariate Cox proportional hazards models were used to investigate the predictors of clinical outcome. Variables that showed an effect on the clinical outcome with a significance level <0.2 in the univariate analysis were entered into the multivariate Cox proportional hazards models. Cox model findings are presented as hazard ratios (HRs) and 95% confidence intervals (CIs). A two-tailed p-value of <0.05 was considered statistically significant.SAS 9.3, SAS Institute Inc., Cary, NC, USA was used for statistical analysis.

## Results

### Population

Table 1 reports clinical characteristics according to the implanted lead. BL group had a slightly lower EF owing to implantation before the 2013 guidelines^11^ release (NYHA 3–4 patients more represented), whereas the AFL patients were on average older, with higher prevalence of old females, AF, diuretic use and comorbidities. All the patients were on optimized medical therapy for heart failure therapy. The leads used in the study period are reported in Table 2. During follow up, CRT delivery was not significantly different across the groups, 97.8% (BL) vs 99% (QL) vs 100% (AFL).

**Table 1:** Study population.

	All	Bipolar leads	Quadripolar leads	Active fixation leads	P^*^
N	290	136	97	57	
Male (n.%)	213 (73.4)	102 (75.0%)	76 (78.4%)	35 (61.4%)	0.16
Mean Age (yrs)	66.3 ± 12.6	63.9 ± 12	67.1 ± 12.2	70.7 ± 13.5	0.08
Ischemic etiology (n,%)	94 (32.4)	45 (33.1%)	32 (33.0%)	17 (29.8%)	0.28
NYHA I-II (n,%)	93 (32.1)	37 (27.2%)	36 (37.1%)	18 (31.6%)	0.19
NYHA III.IV (n,%)	197 (67.9)	99 (72.8%)	61 (62.9%)	39 (68.4%)	0.12
Mean QRS width (ms)	161.8 ± 25.1	161.0 ± 25.5	161.6 ± 23.9	164.1 ± 26.5	0.54
LBBB (n,%)	237 (81.7)	198 (80.1%)	78 (80.4%)	50 (87.7%)	0.81
Permanent AF (n,%)	59 (20.3)	27 (19.9%)	18 (18.6%)	14 (24.6%)	0.77
Mean LV EDV (ml)	226.5 ± 71.5	242.4 ± 75	213.7 ± 64.3	208.6 ± 67.0	0.07
Mean LV ESV (ml)	168.1 ± 59.7	183.0 ± 61.8	156.5 ± 53.5	150.5 ± 56.9	0.08
Mean LV EF (%)	26.0 ± 5.6	24.1 ± 5.2	27.9 ± 5.4	27.8 ± 5.2	0.07
Severe kidney disease (n,%)	24 (8.3)	8 (5.9)	9 (9.3)	7 (12.3)	0.31
Hypertension (n,%)	165 (56.9)	65 (47.8)	61 (62.9)	39 (68.4)	0.01
Hypercolesterolemia (n,%)	142 (49)	57 (41.9)	49 (50.5)	36 (63.2)	0.02
Smoke (n,%)	70 (24.1)	33 (24.2)	27 (27.8)	10 (17.5)	<0.001
Diabetes (n,%)	71 (24.2)	31 (22.3)	27 (27.8)	13 (22.8)	0.64
Beta-Blockers (n,%)	261 (90.0%)	124 (91.2%)	87 (89.7%)	50 (87.8%)	0.83
ACE-I/ARB (n,%)	238 (82.1%)	114 (83.8%)	80 (82.5%)	44 (77.2%)	0.91
Diuretics (n,%)	263 (90.7%)	121 (89.0%)	88 (90.7%)	54 (94.7%)	0.90
Potassium-sparing (n,%)	168 (57.9%)	75 (55.1%)	57 (58.8%)	36 (63.2%)	0.51
Average Follow up (months)	12	12	12.1	10.4	0.91

^*^Chi square test performed.

Legend: LBBB: left bundle branch block; AF: atrial fibrillation; LV: left ventricular; EDV: end diastolic volume; ESV: end systolic volume; EF: ejection fraction.

**Table 2:** LV leads employed in the study population.

Left ventricular leads	Manufacturer	Model	N (%)
Bipolar	Boston Scientific	Easy Track 3 4548	41 (14)
	Medtronic	Ability 4296,4196	66 (23)
	St. Jude Medical	Quicksite 1056T	29 (10)
Quadripolar	Boston Scientific	Acuity X4 4677	1 (0.3)
	Medtronic	Performa 4298	72 (25)
	St. Jude Medical	Quartet 1458Q	24 (8)
Active fixation	Medtronic	Stability 20066	57 (20)

### Technical composite endpoint

**Figure 2:**
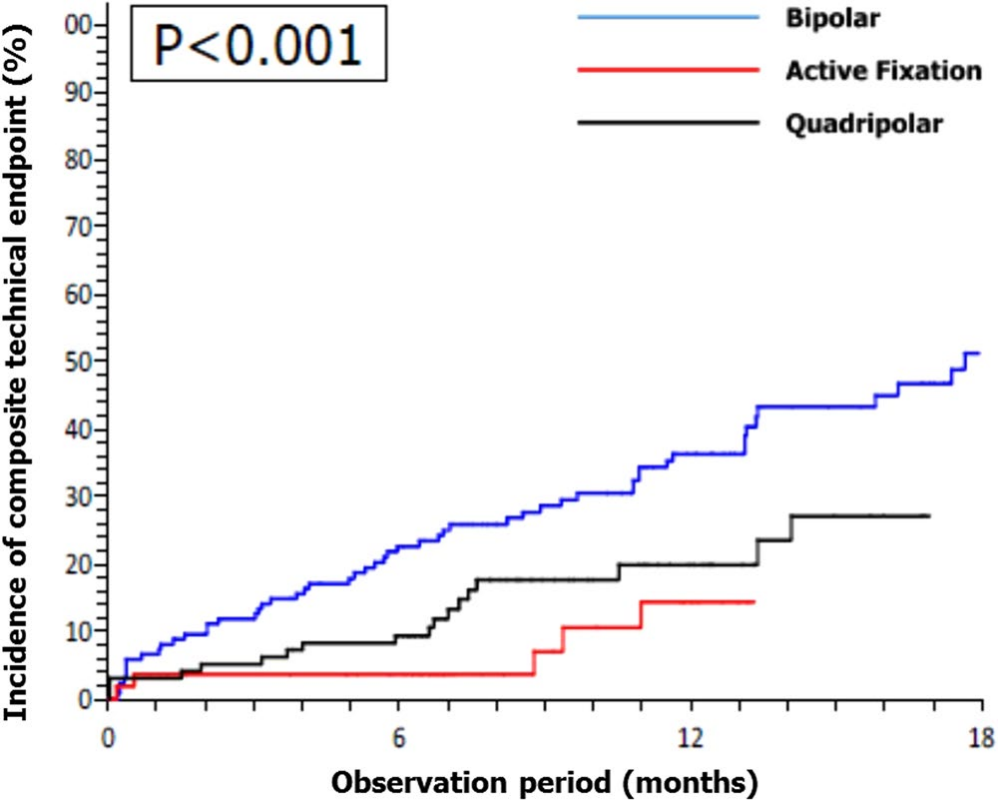
Composite technical endpoint with different left ventricular leads.

The incidence of composite technical endpoint at long term follow up is reported in Table 3 and Fig. 2; overall TE occurred in 27.5%, but did not prevent CRT delivery in any patient during follow-up. Dislodgment requiring re-operation occurred in 10 (7.4%) BL patients, in 4 (4.1%) QL patients (2 with a Quartet St. Jude Medical leads and 2 with Performa Medtronic leads), and in no AFL patient. Five of these patients (4 with a BL and 1 with a QL) could no longer be paced in the same targeted site because of coronary vein occlusion (after the LV lead dislodgement).

**Table 3:** Technical and Clinical Endpoints.

	All	Bipolar leads	Quadripolar leads	Active fixation leads	P^*^
**Technical Endpoints**					
PNS > 8 V @ 0.4 ms (n,%)	33 (11)	23 (17)	8 (8)	2 (4)	0.014
Safety Margin between PN and LVT threshold <2 V (n,%)	24 (8)	19 (14)	2 (2)	3 (5)	0.003
Failure to achieve TPS (n,%)	26 (9)	22 (16)	3 (3)	1 (2)	<0.001
LV Lead dislodgment (n,%)	27 (9)	21 (15)	5 (5)	0(0)	0.003
LV Lead dislodgement requiring a re-operation	14 (4.8)	10 (7.4)	4 (4.1)	0(0)	0.005
**Clinical Endpoints**					
Death for any causes (n,%)	18 (6.2)	10 (7.3)	5 (5.2)	3 (5.3)	0.75
Hospitalization for heart failure (n,%)	26 (9)	17 (12.5)	7 (7.2)	2 (3.5)	0.24
Heart Transplantation (n,%)	3 (1)	2 (1.5)	1 (1)	0	0.65
Infection leading to lead extraction	^2 (0.7)^	^1 (0.7)^	^1 (1)^	^0^	0.84

*Chi square test performed.

Legend: PNS: phrenic nerve stimulation; LVT: left ventricular threshold; TPS: target pacing site; LV: left ventricular.

**Figure 3:**
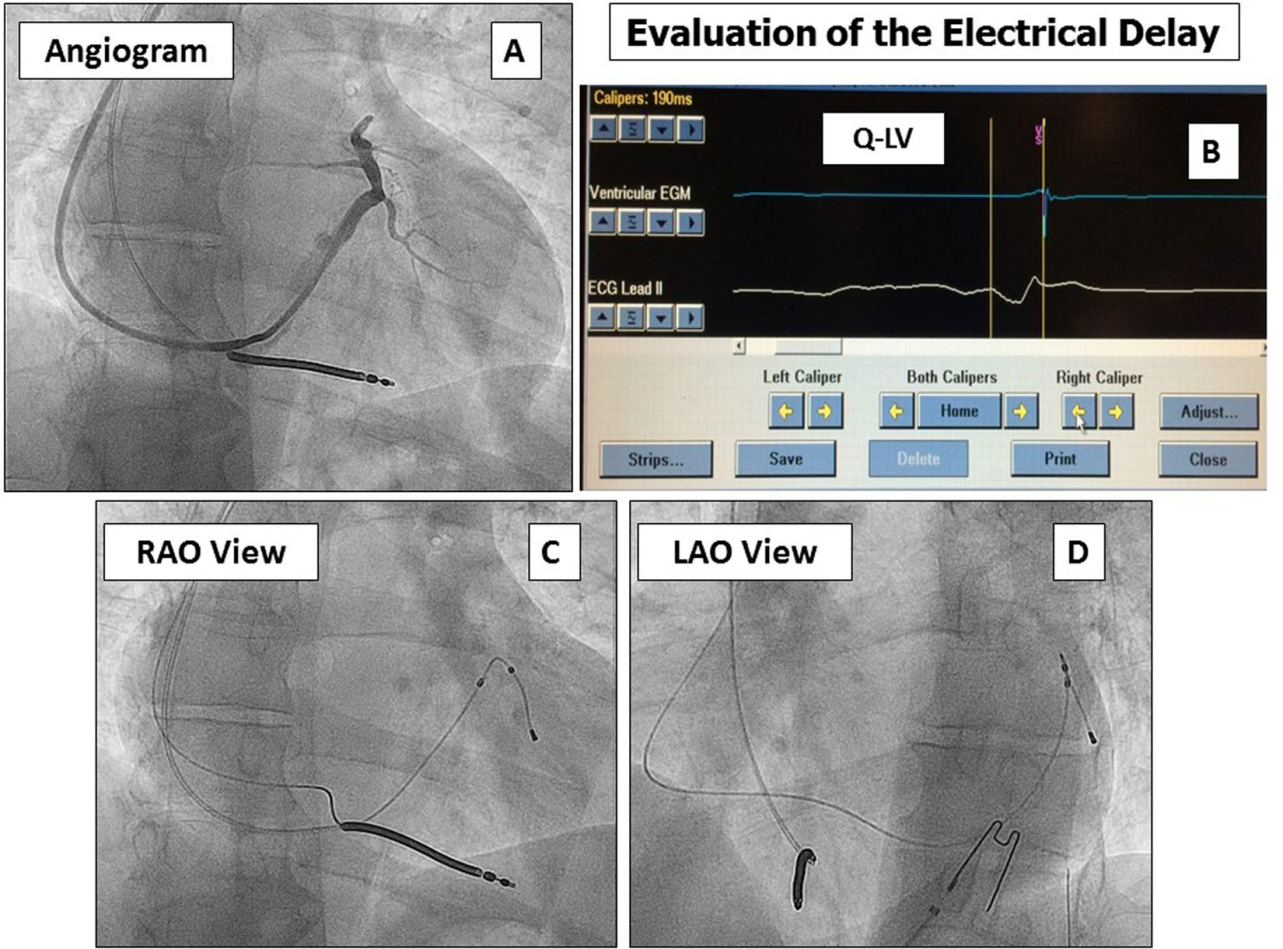
Coronary sinus angiogram and target pacing site evaluation. Panel A: coronary sinus angiogram that showed a lateral short vein; panel B: Q-LV methods for the electrical delay evaluation^9^; panel C: final left ventricular lead position in right anterior oblique (RAO) view; panel D: final left ventricular lead position in left anterior oblique (LAO) view.

Significant differences in the composite TE were observed amongst the 3 LV lead groups: QL enabled superior management of PN, whereas AFL proved superior for LV targeting and prevention of dislodgment (Table 3, Fig. 2) regardless of a difficult coronary vein anatomy and right atrial enlargement (Fig. 3).

### Composite clinical endpoint

**Figure 4:**
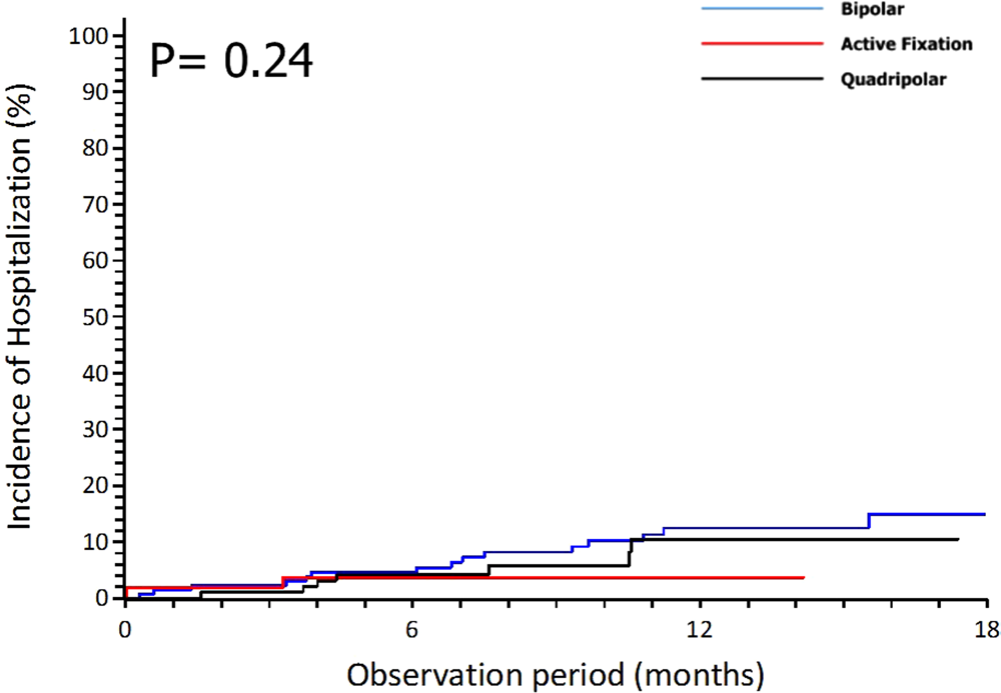
Hospitalization for heart failure with different left ventricular leads.

The incidence of composite clinical endpoint at 12 months follow up was 10.3% without significant differences between the 3 groups (p = 0.38) (Table 3, Fig. 4). Infections leading to system removal occurred in 2 pts (one in BL group and the other in the QL group) after respectively 8 and 6 months from implantation (Table 3).

### Reverse remodeling

**Figure 5:**
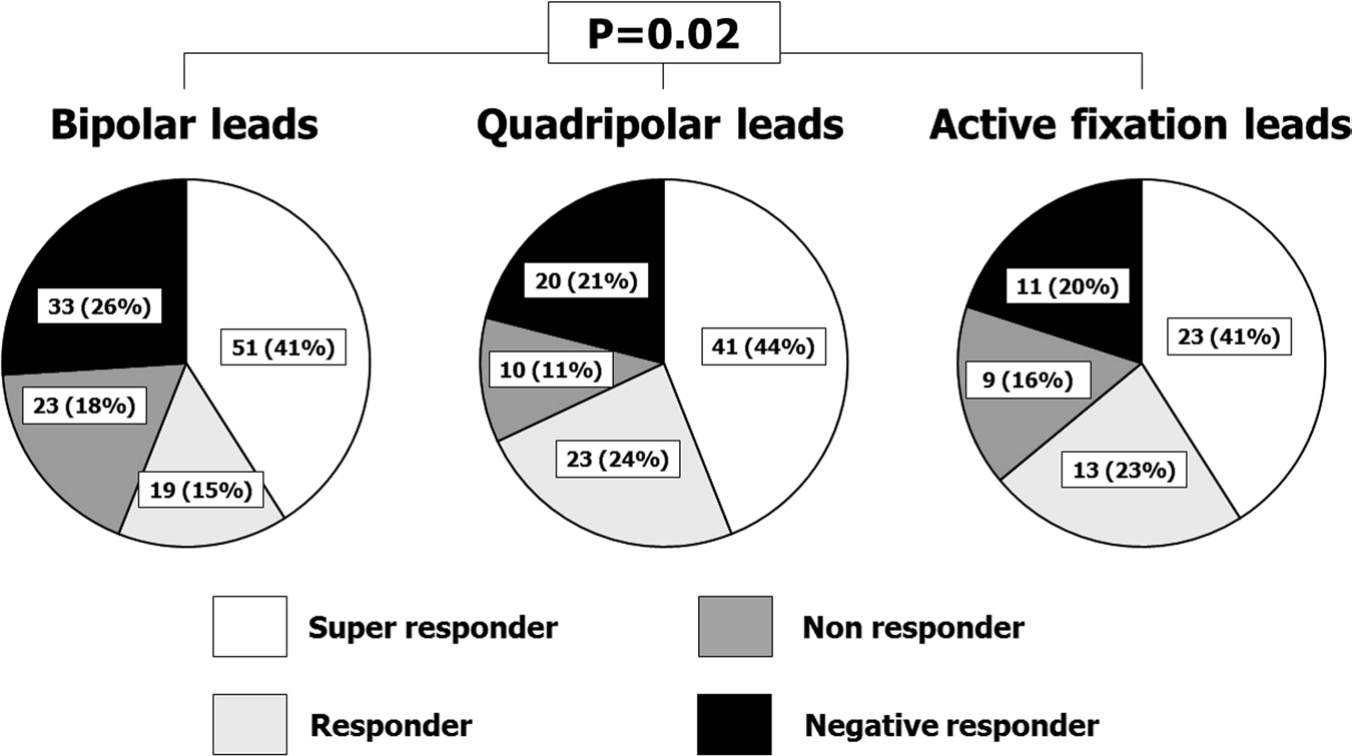
Reverse remodeling and quality of response^11^ by 10-months Echocardiography.

At follow up 170/270 (63%) living patients had reverse remodeling (reduction of ESV > 15%), but there were differences in the 3 LV lead groups (Fig. 5): responders were 56%, 68%, 64% respectively in BL, QL and AFL (p = 0.02). No significant differences occurred between QL and AFL. On a multivariable regression analysis, only “non-BL leads” were associated to an improved clinical outcome (reverse remodeling + free from heart failure related hospitalizations/death/heart transplantation; see Table 4).

**Table 4:** Reverse Remodeling + free from HF related hospitalizations/death/heart transplantation.

Variables	Odds Ratio (Low 95% CI-High 95% CI)	P value
**Univariate analysis**		
Age	1.00 (0.98–1.02)	0.735
Male	1.09 (0.65–1.84)	0.743
Non ischemic Etiology	1.43 (0.87–2.35)	0.155
Diabetes	0.91 (0.53–1.56)	0.732
Kidney disease (VFG < 30 ml/min)	0.58 (0.25–1.38)	0.218
LBBB	1.59 (0.87–2.93)	0.135
150 < QRS < 170	1.05 (0.65–1.71)	0.835
QRS > 170	0.99 (0.60–1.62)	0.962
Baseline EF < 20%	0.65 (0.31–1.37)	0.260
ESV/BSA < 43 ml/m^2^*	2.74 (0.54–13.81)	0.223
Achieving TPS	1.66 (0.73–3.80)	0.228
Non bipolar lead	2.02 (1.26–3.22)	0.003
Multivariate analysis**		
Male	1.31 (0.76–2.27)	0.333
Non ischemic Etiology	1.56 (0.93–2.62)	0.089
LBBB	1.71 (0.91–3.22)	0.095
Non bipolar lead	1.99 (1.24–3.20)	0.004

^*^BSA calculated with Mosteller’s Formula √[(height*weight/3600)].

^**^Only parameters with a p value < 0.200 and demographic characteristics where included in the multivariate analysis.

Legend: LBBB: left bundle branch block; ESV: end systolic volume; BSA:body surface area; TPS: target pacing site.

## Discussion

This is an observational cohort, not a randomized clinical trial with the inherent limitation (as described in the dedicated section) but representing the real world. Our study highlights that QL and mostly AFL enable an easier targeted LV lead placement compared to traditional BL, with a trend in favor to less heart failure-related hospitalizations. Owing to superior stability coupled with small size, trackability along tortuous veins and steroid elution on either tip or ring electrode, the Attain Stability^TM^ proved superior in overcoming the main challenges to LV lead implantation at a targeted placement site regardless of coronary vein anatomy and risk of dislodgement. In fact, short and thin veins as long as 3 cm could be considered for LV lead stimulation (Figs 1 and 3). Targeted LV stimulation has indeed demonstrated to increase the chances of RR and clinical improvement following CRT implantation^12^.

### Technical Endpoint and LV leads

PN, LV lead dislodgement and high LVT prevent a stable and targeted CRT delivery along follow up, thus creating the background for CRT-nonresponse^2–6,13,14^. New multipolar leads enable an easier management of PN and broader possibilities to reach the target stimulation site. Despite this, the LVT threshold at the target location may not differ from one lead platform to the other, being dependent on local tissue properties, electrode technology, and stabilization strategy^2–6,13,15^. Whereas QL leads ensure an easier management of PN and fewer lead dislodgments compared to conventional BL leads in comparative studies^16^, the dislodgement rate of QL was 3.5% at 3 months in Tomassoni multicenter study^5^, and very similar at one year in this study (with some differences across manufacturers). Enhanced stability portends improved clinical outcome: Forleo *et al*.^16^ observed a reduction of heart failure-related hospitalizations and of LV lead dislodgements by QL compared to conventional BL, although the study was not randomized and no strategy for coronary vein selection was used for both type of leads. Being RR data not reported in that study^16^, it is likely that the clinical outcome has been influenced also by factors other than the QL technology, that is the TE may not be related to the CE. In our study, the strategy aimed at the latest activated site of the accessible veins at least 5 F in size in all the patients, CRT delivery was optimized and delivered at the same extent in all the patients, hence the incidence of TE, the RR extent and the CE can be associated to lead technology.

### Clinical Endpoint

Resynchronization therapy is a customized therapy, targeted pacing site being a key point for LV reverse remodeling and long-term outcome^12^. LV lead placement at a scar-free site^8,17^ possibly mechanically^12^ or electrically delayed (Fig. 3 panel B) improve RR up to 70% of patients. Avoidance of LV dislodgement over the first year after implantation is hence of pivotal importance, since displacement has been reported to reach a ceiling after 10 months^11,14,15^. Under this perspective, the use of AFL enhances the capability of targeted LV lead placement^4^ while ensuring stability over the long term (Table 3), possibly improving the clinical outcome (Table 3). This is particularly important as only 35% of patients have more than a single coronary vein suitable to LV lead placement^6^, and QL leads may not be successfully implanted in up to 7% of patients^17,18^. Despite these considerations in our study the “novel technologies”, while significantly increasing RR, failed to show an improvement in clinical composite score. The reason for this result presumably lay in study design which compared three active strategies for CRT (and not a placebo/drug-only arm) that may require longer follow-up time to show superiority in terms of survival/hospitalization. However, in view of the close relationship between RR and clinical outcomes we believe that future studies with larger cohorts and longer follow-up will obtain positive results also in terms of hospitalization reduction and even survival. One possible limitation of bipolar AFL could be the impossibility to elicit multi-point LV stimulation at inter-electrode intervals broader than 20 mm; this novel technology awaits to prove superiority in non-responders to single site LV stimulation in the randomized More-CRT MPP study (Clinical Trials.gov Identifier NCT02006069), and has been shown to have a highly individual efficacy on acute hemodynamic measurements, when a strictly pathophysiologic approach is applied^19^. Some concern could be raised about AFL extractability in the event of CIED infection: the conception of the Attain Stability^TM^ lead is such to minimize the labor of lead extraction, and although leads being in place for several years have to be challenged, we previously reported an uneventful extraction procedure in a patient (not included in this population being a CRTP recipient) with a device infection implanted with an AFL lead^20^.

## Limitations

This is a single center, retrospective non-randomized study that reflects the clinical practice in CRT at an experienced teaching hospital in consecutively implanted patients. The population is outnumbered for mortality endpoints, and a longer follow up could strengthen our argumentation. Moreover, the potential confounders in the assessment of CE were even among the study groups owing to a similar care in device tailoring and CRT delivery along follow up. Finally we must remember that measuring RR with echocardiography is challenging and shows important inter-observer variability even if our inter-observer variability is quite good^10^.

## Conclusions

Left ventricular QL and AFL enable a superior RR compared to conventional BL, owing to a decreased dislodgement rate from the targeted stimulation site and freedom from other technical challenges^21^. AFL and QL solve the same technical issues, but in different way, the first one with the active fixation and the second one with electronic repositioning. Both new technologies permit a targeted pacing, then AFL is the best lead to prevent dislodgement instead QL is the best for the PN management.

## Data Availability

The datasets generated during the current study are not publicly available due to restrictions claimed by the ethical committee but are available from the corresponding author on reasonable request and formal request to the local ethical committee.
